# Determination of DTaP vaccine potency by multiplex immunogenicity testing using electrochemiluminescence

**DOI:** 10.1038/s41541-024-00915-y

**Published:** 2024-08-07

**Authors:** Bärbel Friedrichs, Simone Rehg, Kay-Martin Hanschmann, Volker Öppling, Isabelle Bekeredjian-Ding

**Affiliations:** 1 Paul-Ehrlich Institut, Federal Institute for Vaccines and Biomedicines, Paul-Ehrlich-Strasse 51-59, D-63225 Langen, Germany; 2 Present Address: Institute for Medical Microbiology and Hospital Hygiene, Philipps-University Marburg, Hans-Meerweinstr. 2, D-35043 Marburg, Germany

**Keywords:** Protein vaccines, Protein vaccines

## Abstract

Lot release testing of diphtheria, tetanus and acellular pertussis vaccines traditionally relied on in vivo protection models involving challenge of laboratory animals with toxins. Meanwhile, many labs have switched to serological testing of these vaccines, which is often performed in separate in vivo assays, even if all components were formulated into one vaccine product. Here we describe the results of simultaneous serological potency determination of diphtheria (D), tetanus (T) and acellular pertussis (aP) antigens obtained following immunization of guinea pigs with multicomponent pediatric and booster vaccines from different manufacturers. The 4th World Health Organization (WHO) International Standard (IS) for diphtheria toxoid (No. 07/216) and the 4th WHO IS for tetanus toxoid (No. 08/218) were used as reference preparations. For aP, a pediatric vaccine batch containing the antigens pertussis toxoid, filamentous hemagglutinin, pertactin and fimbriae proteins type 2/3 was established as internal control. Quantification of IgG against D, T and aP antigens in guinea pig sera was performed using a hexaplex electrochemiluminescence immunoassay. We further provide proof-of-concept using experimental vaccine samples lacking or containing reduced amounts of diphtheria toxoid in the presence of full amounts of tetanus and pertussis antigens and alum adjuvant. Importantly, the assay confirmed dose-response relationships for all antigens tested and was able to detect diphtheria out-of-specification batches. The results confirmed the suitability of the protocol for combined serology batch release testing of DTaP combination vaccines as first measure towards implementation of full in vitro testing of DTaP vaccines. This report summarizes the data and the protocol used for validation prior to implementation of this method in routine batch release testing of DTaP vaccines, which led to replacement of in vivo challenge experiments in our laboratory following the 3 R (replace, reduce, refine) principle.

## Introduction

Vaccines are essential tools for preventing severe diseases and deaths induced by infectious agents. Hence, maintaining the quality of vaccines is required, and has historically led to confirmatory testing and batch release by governmental authorities.

Currently, substantial effort is underway to develop in vitro tests to substitute for animal experiments in safety and potency batch release of vaccine products. Additionally, the quality control procedures for many vaccines—in particular for more recent vaccine licensures—consist of solely in vitro, e.g. non-animal assays^1^. However, batch release testing of diphtheria (D), tetanus (T) and acellular pertussis (aP) vaccines is still performed in animals. Notably, these vaccines are among the most frequently administered vaccines worldwide because they are key components of all childhood immunization programs and used to boost immunity in adolescents and adults^2,3^. For estimation of potency of adsorbed tetanus and diphtheria vaccines, many test laboratories adhere to challenge experiments with lethal endpoints with the consequence of severe distress in a large number of animals. However, in the interest of animal welfare, a series of collaborative studies were conducted in the European Union, that resulted in revisions of the European Pharmacopeia monographs on diphtheria and tetanus potency testing to include a recommendation to use serological assays instead of challenge tests for routine batch release^2,4,5^.

Quantification of serum antibody levels has historically been used in both animals and humans to diagnose ongoing and past exposure to infectious pathogens for treatment or surveillance purposes, to monitor therapeutic interventions in chronic infections such as borreliosis or to provide proof of vaccination-induced protection on individual and population levels. In the case of tetanus and diphtheria, the immune correlates of protection are well defined^6^, e.g., neutralizing antibody titer values of 0.01–0.1 International Units (IU)/ml for diphtheria and 0.1 IU/ml for tetanus, and can be used for decision-making on revaccination. In views of this clinical use of toxin-neutralizing antibody titers it is not surprising that the acceptance of an in vivo model for potency detection continues to be high. However, the animal model does not necessarily reflect the human immune response and high variability encountered in animal testing is a continued challenge^7,8^.

For many decades, enzyme-linked immunosorbent assay (ELISA) represented the standard readout method to quantify disease- or antigen-specific serum antibodies. Meanwhile, various multiplex testing platforms, such as bead-based flow cytometric assay and electrochemiluminescence (ECL) immunoassay (ECLIA) have been developed, which have not only led to the decreased sample and reagent volume needed but also to an increased testing throughput^9–16^. Furthermore, multiplex assays were shown to perform at least similarly or even better with regards to sensitivity and reproducibility of antibody quantitation^10,17–20^. ECL detection provides many advantages, in particular ultra-high sensitivity and broad dynamic range because of signal amplification by multiple excitation cycles, low background because of decoupling of stimulation method (electricity) from signal (light) and great flexibility since Sulfo-TAG labels are stable, non-radioactive and can conveniently be conjugated to biological molecules^10^. The development of multiplex immunoassays can be challenging due to factors such as cross-reactivity of antibodies and antigens or a short range of linearity despite a broad range of signals, i.e., analyte concentrations to be measured in the samples. We reasoned that an ECLIA, which possesses a wide linear detection range could be a suitable choice for simultaneous measurement of different serum antibodies following immunization with multi-component vaccines in the presence of a blocking buffer suppressing any unspecific cross-reactions.

Previous studies already indicated that the guinea pig is a suitable animal model for serological assays since dose-response relationships for several combination vaccine antigens lay in the same range^2,21^. Since DT vaccines are increasingly produced in formulations that additionally contain pertussis antigens, which require their own in vivo test for immunogenicity, we sought to develop a method that allows to test all components (D, T and aP) in one serology test protocol and thereby reduce animal numbers and animal distress^22^. Using a pediatric and booster vaccine product and ECLIA for serum antibody detection the present study confirmed that combined serological analysis of D, T, and aP components is feasible in guinea pigs.

## Results

### Electrochemiluminescence immunoassays provide a reliable and precise technology for simultaneous detection of antibodies against DTaP antigens

A hexaplex ECLIA was developed for quantitation of serum IgG against the tested vaccine antigens, i.e., diphtheria toxoid, tetanus toxoid and four aP antigens (pertussis toxoid [PT], filamentous hemagglutinin [FHA], pertactin [PRN] and fimbriae proteins type 2/3 [FIM]).

**Fig. 1:**
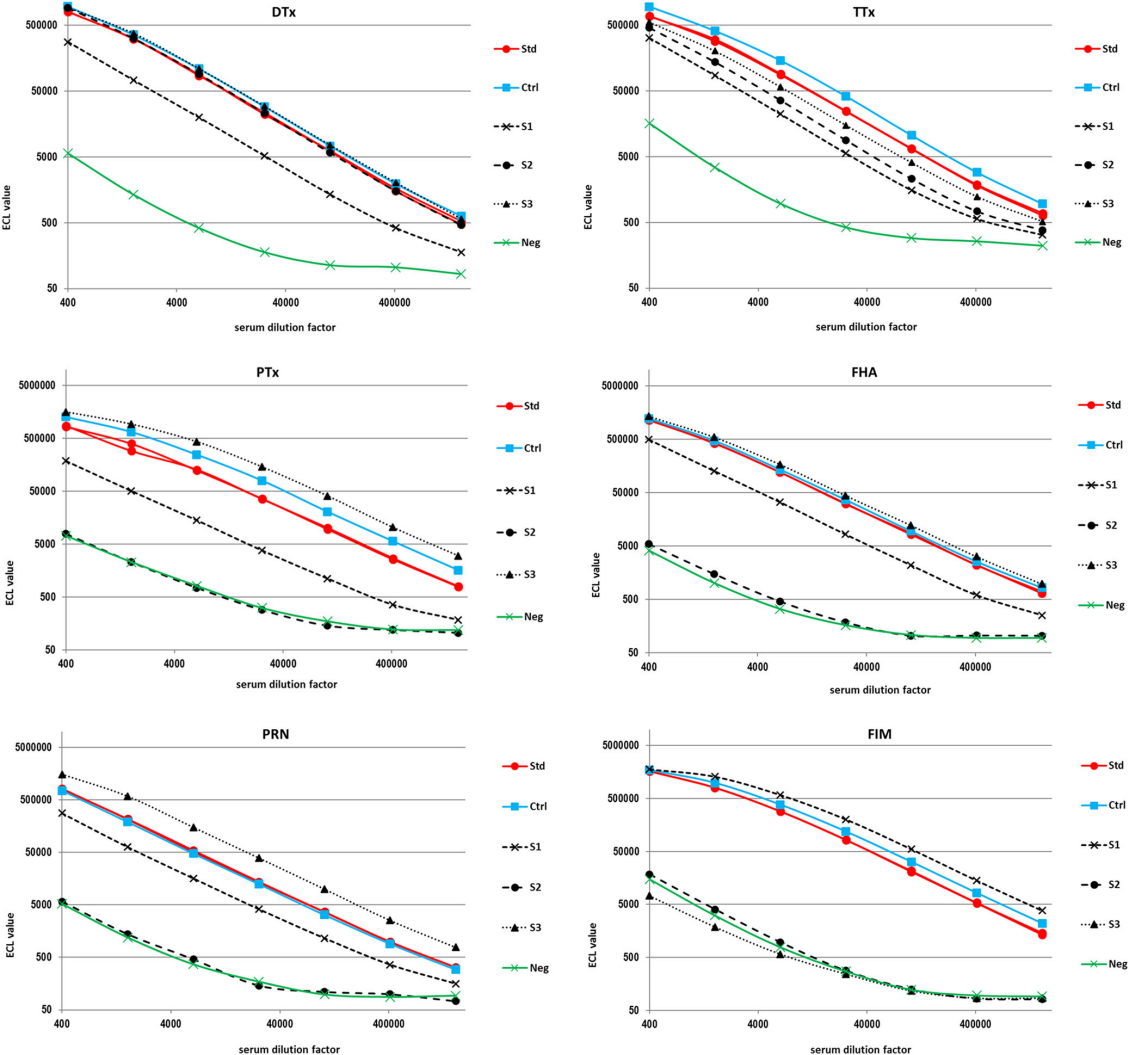
Titration of standards and assay controls. Titration curves of standard (Std), positive (Ctrl) and negative (Neg) control sera as well as individual test sera from immunized guinea pigs (S1, S2, S3), starting with dilution 1:400. ECL results obtained on the various antigen spots (DTx, TTx, PTx, FHA, PRN and FIM) are depicted.

Figure 1 depicts a representative assay with three test sera (S1, S2, S3). S1 was obtained from a guinea immunized with a dtap vaccine batch, S2 was obtained following immunization with reference vaccines Diphtheria Toxoid Adsorbed and Tetanus Toxoid Adsorbed; S3 following immunization with a pediatric DTaP-HB-IPV vaccine batch. Additionally, a qualified standard serum and a positive and a negative control serum were applied to the plate. All sera were used at a starting dilution of 1:400 and serially diluted 1/4 over 6 dilution steps (1/400 to 1/1638400) (Fig. 1). Using the parallel-line model, IgG specific for each of the six antigens in each test serum was calibrated against the standard using at least three consecutive dilution points per serum. Similarly, positive and negative controls were quantified; for QC purposes, results for both controls must meet specification limits for each assay plate in order to validate the assay.

**Fig. 2:**
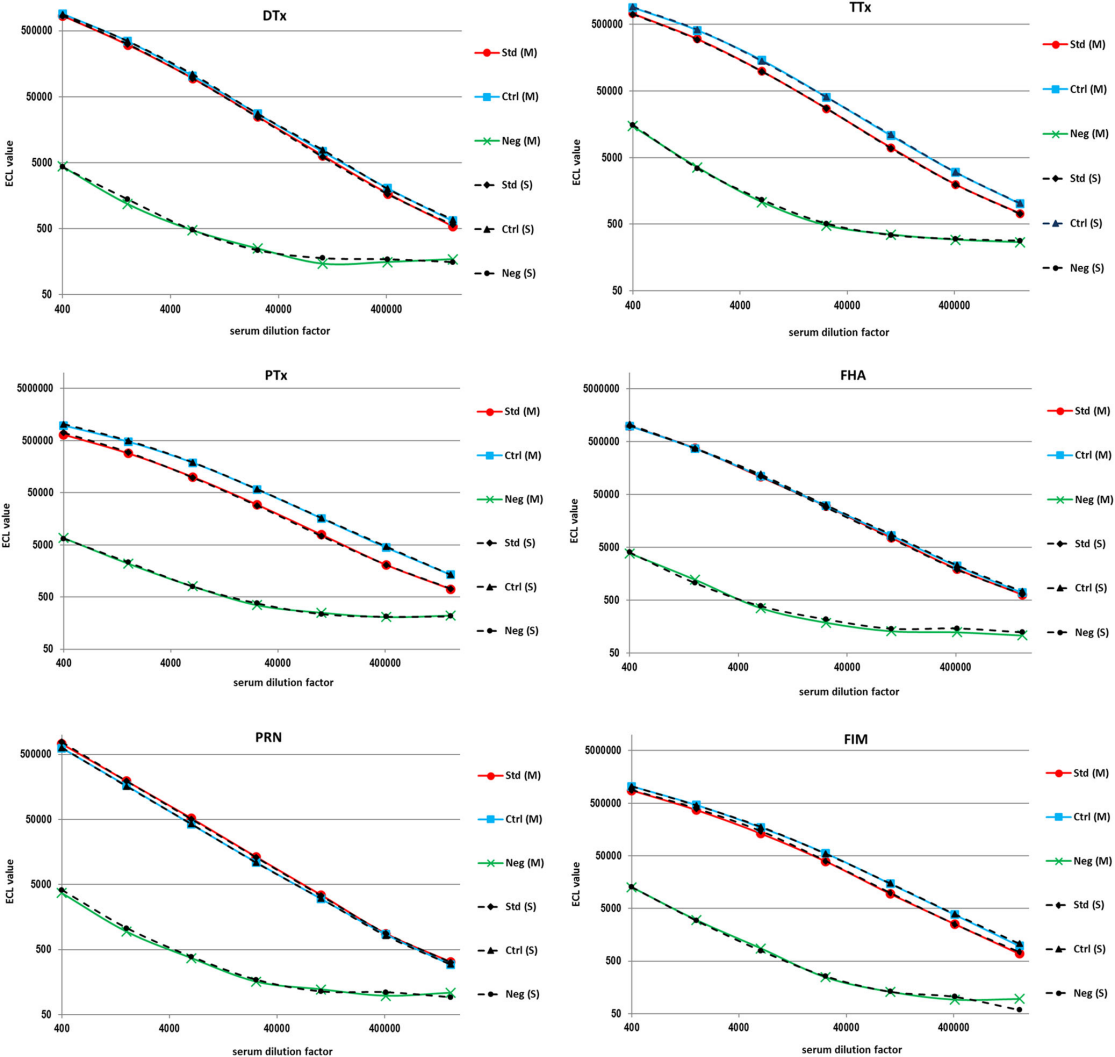
Comparison of titration curves obtained for multiplex (M) and singleplex (S) immunoassays using standard (Std), positive (Ctrl) and negative (Neg) control sera. ECL results obtained on respective antigen spots (DTx, TTx, PTx, FHA, PRN or FIM) are depicted.

To demonstrate that multiplex results are not impaired by cross-reactivity of serum antibodies to any unrelated antigen, multiplex analyses were compared to separate singleplex analyses using a single antigen coupled to its specific linker only, e.g., linker 1-DTx or linker 3-PTx, leaving the residual 5 linkers uncoupled. Initial experiments showed that singleplex assays with usage of only one linker-coupled antigen resulted in unspecific binding of serum components to the plate spots and thus background ECL signals. This could be avoided by adding all uncoupled linkers (i.e., without antigen). As depicted in Fig. 2, multiplex and singleplex dilution curves of standard and control sera align almost identical for each of the six antigens. Thus, in the multiplex setup, cross-reaction of guinea pig serum antibodies with unrelated antigens which might impact binding to its specific antigen (-spot) could be excluded. Specificity of the multiplex assay was partially determined indirectly. ECL values for ‘specific’ IgG lay in the range of those measured with negative serum when vaccine formulations lacking respective antigens were used for immunization. E. g., sera obtained from diphtheria and tetanus reference vaccine groups, lack antibodies against pertussis antigens (Fig. 1, S2). Correspondingly, in sera obtained following immunization with an DTaP-HB-IPV vaccine containing PT, FHA, and PRN IgG against PT, FHA, and PRN are detected, whereas anti-FIM IgG were missing (Fig. 1, S3).

Precision was assessed by calculation of intra- and inter-assay variation and total measurement uncertainty after having performed the protocol 21 times (17 assays with four plates each and 4 assays with one plate) under standard conditions by two technicians on different experimental days. Intra-assay and inter-assay variation coefficients for positive control serum antibody levels ranged from 5.7 to 8.6%, and total measurement uncertainty from 8.0 to 10.0% for all IgG concentrations measured (Table 1).

**Table 1:** Coefficient of variation and measurement uncertainty of the multiplex ECLIA

IgG antibody against	Coefficient of variation intra assay	Coefficient of variation inter assay	Measurement uncertainty
Diphtheria toxin	7.2%	7.3%	8.7%
Filamentous hemagglutinin	6.0%	7.0%	8.0%
Fimbriae proteins type 2/3	6.9%	7.5%	8.8%
Pertactin	5.7%	8.5%	9.3%
Pertussis toxin	8.6%	8.2%	10.0%
Tetanus toxin	6.4%	7.4%	8.5%

Determination of IgG concentration in positive control serum by calibration against standard serum in a total of 72 assays comprising 4 assays on one plate and 17 assays on 4 plates performed in parallel.

### Immunization with diphtheria and tetanus toxoids induces IgG in a dose-dependent manner

Serum IgG concentrations were measured in sera obtained 35 days after immunization with reference vaccines in nine repeat experiments. Supplementary Fig. 2 shows the results obtained from guinea pigs immunized with 4th WHO IS Diphtheria Toxoid and 4th WHO IS Tetanus Toxoid at three different doses. Although high variability of IgG concentrations was observed, diphtheria (Supplementary Fig. 2a) and tetanus (Supplementary Fig. 2b) IgG levels showed a reproducible dose-response relationship.

**Fig. 3:**
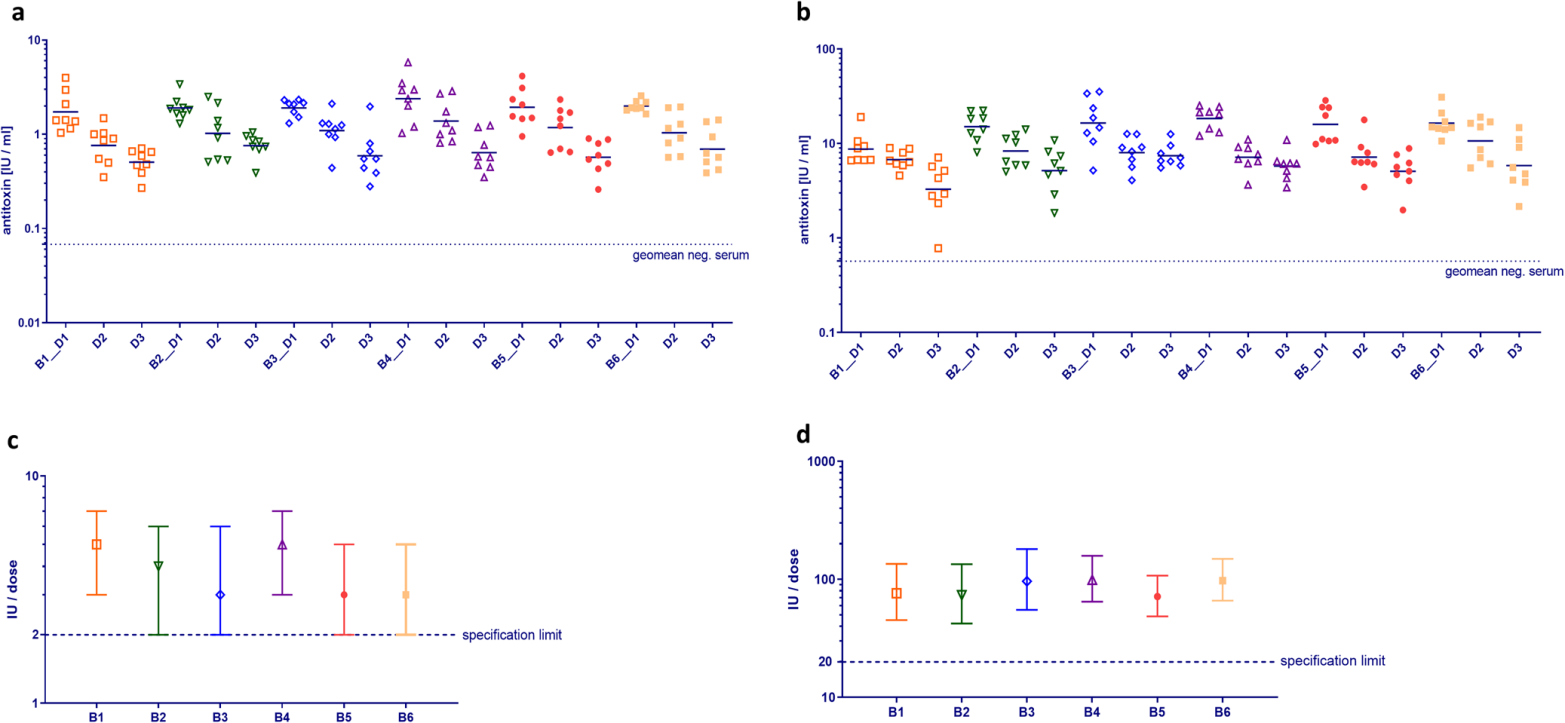
Immunogenicity and potency of the diphtheria and tetanus component in dtap booster vaccines. Immunogenicity of diphtheria (**a**) and tetanus (**b**) component of six batches of a dtap booster vaccine in guinea pigs. Vaccine batches (B1–B6) were applied at 3 dose concentrations in separate animal studies. D1 = 1/1, D2 = 1/3, D3 = 1/9. Each symbol depicts the IgG/antitoxin response of one individual guinea pig (8/dose); bars: geomean of antibody response per dose. Dotted line: geomean of naïve guinea pigs (negative control serum). Diphtheria (**c**) and tetanus (**d**) potency (estimate ±95% CI) obtained for dtap vaccine batches by calibration against 4th WHO IS Diphtheria Toxoid Adsorbed (07/216) and 4th WHO IS Tetanus Toxoid Adsorbed (08/218), respectively. All batches met the specification limits (dotted lines) of 2 IU/dose for diphtheria toxoid and 20 IU/dose for tetanus toxoid at the LL of 95% CI.

Similar dose-response relationships were found in animals previously immunized with combination vaccines against diphtheria, tetanus and pertussis (acellular). Figure 3 depicts diphtheria (Fig. 3a) and tetanus (Fig. 3b) IgG responses in guinea pigs immunized with six batches (B1–B6) of a commercial dtap booster vaccine product.

### ECLIA results confirm potency of diphtheria and tetanus toxoids in multivalent vaccine products

As explained above the aim of batch release testing is to ensure consistency and potency of vaccines. It was therefore key to evaluate whether immunization-induced IgG measured by ECLIA can be used as a potency indicator for all types of vaccines.

#### Booster vaccine with reduced antigen content

Diphtheria and tetanus potency of individual vaccine batches was calculated relative to the potency of the respective reference preparation (4^th^ WHO IS Diphtheria Toxoid Adsorbed or 4th WHO IS Tetanus Toxoid Adsorbed) applied within the same animal study using the parallel line model. Diphtheria and tetanus potency obtained for the individual batches of the dtap vaccine are depicted in Fig. 3c, d, respectively. According to European Pharmacopeia^23^, human vaccines indicated for booster vaccination of children and adults must contain at least 2 IU/dose of diphtheria toxoid and 20 IU/ dose of tetanus toxoid at the LL of 95% CI.

All batches of dtap vaccine depicted in Fig. 3c passed the diphtheria potency test with an estimate of 3–5 IU/dose and a LL of 95% CI of 2–3 IU/dose.

When the same batches of dtap vaccine were analyzed for tetanus potency relative to the respective reference preparation of tetanus toxoid, they contained a calculated amount of 72–99 IU/dose tetanus toxoid with a LL of 95% CI between 42 and 66 IU/ dose (Fig. 3d), which is above the minimum amount needed to meet the specification (i.e., 20 IU/dose).

#### Pediatric vaccine for primary immunization

**Fig. 4:**
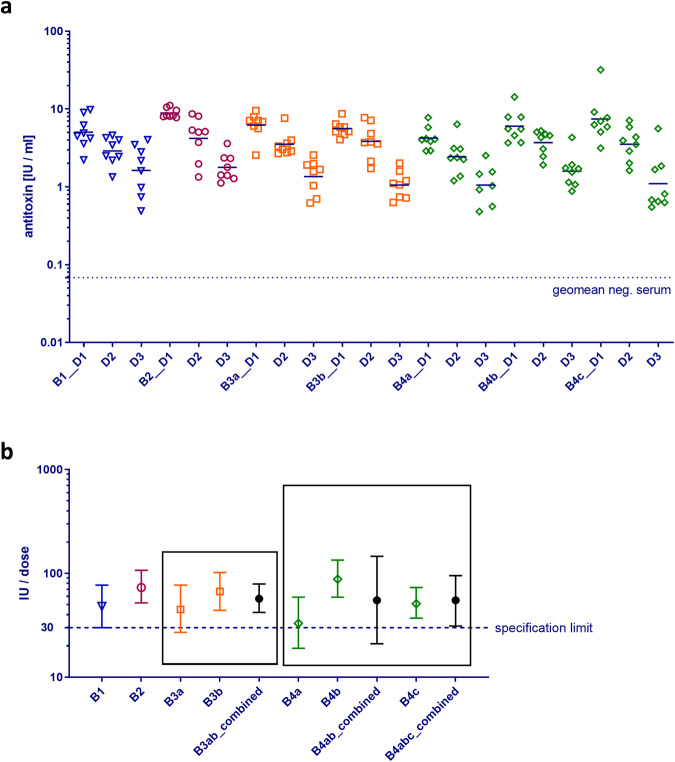
Immunogenicity and potency of the diphtheria component DTaP pediatric vaccines. **a** Immunogenicity of diphtheria component of four batches of a DTaP pediatric vaccine in guinea pigs, partly following repeat animal testing. Vaccine batches (B1–B4) were applied at 3 dose concentrations with batches B3 and B4 applied repeatedly (B3a, B3b or B4a, B4b, B4c). D1 = 1/3, D2 = 1/9, D3 = 1/27. Each symbol depicts the IgG/antitoxin response of one individual guinea pig; bars: geomean of antibody response per dose (8 guinea pigs/dose). Dotted line: geomean of naïve guinea pigs (negative control serum). Note that for dose group B4a_D3 only 7 animals were available. **b** Diphtheria potency (estimate ±95% CI) obtained for DTaP pediatric vaccine batches by calibration against 4th WHO IS Diphtheria Toxoid Adsorbed (07/216). Batches B3 and B4 did not meet the specification limit of 30 IU/dose of diphtheria toxoid (LL of 95% CI, dotted line) in the first animal test (B3a, B4a, respectively). Repeat testing resulted in a ‘pass’ result following combined evaluation of 2 tests for batch B3 (B3ab_combined) and 3 tests for batch B4 (B4abc_combined).

Diphtheria and tetanus potencies of pediatric (DTaP) vaccine batches were calculated as described for booster vaccines. IgG responses against tetanus toxoid and thus tetanus potency was generally well above the minimum amount required, i.e., 40 IU/dose (LL of 95% CI)^24^ (Supplementary Fig. 3). By contrast, formulation of the diphtheria component in pediatric vaccines proved to be in the range of the minimum amount, i.e., 30 IU/dose (LL of 95% CI). Of note, this was in line with the observation that animal experiments frequently resulted in an initial ‘fail’ result for diphtheria potency of pediatric vaccine batches, i.e., below 30 IU/dose at the LL of 95% CI (Fig. 4a, b). In accordance with the relevant European Pharmacopeia monograph^4^, it was necessary to repeat the experiment and perform combined evaluation of test results. As depicted in Fig. 4b, after repeat testing DTaP batches generally passed the diphtheria potency test in the combined assay (Fig. 4b, B3ab_combined). However, one batch (B4) showed an inhomogeneity of results between both tests (B4a and B4b) at *p* < 0.10 so that a semi-weighed combination of results was to be applied, which resulted in a wide CI for the combined result (Fig. 4b, B4ab_combined) and thereby an overall ‘fail’ result (21 IU/dose, LL 95% CI) although the estimate of diphtheria potency was calculated at 51 IU/dose. After a third animal testing of this batch, the overall combination of all 3 tests (B4abc_combined) finally resulted in a pass result for diphtheria potency at 31 IU/dose (LL of 95% CI; estimate 55 IU/dose) (Fig. 4b).

### ECLIA results confirm immunogenicity of acellular pertussis vaccine components

**Fig. 5:**
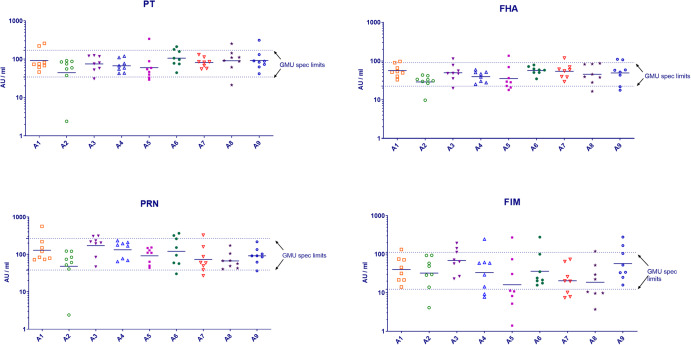
Immunogenicity of acellular pertussis components of aP Ctrl vaccine batch applied in nine separate animal studies (A1 to A9) at single-dose (1/3 dilution). Each symbol depicts the antibody response of one individual guinea pig against the respective aP antigen (8 guinea pig sera/dose); bars: geomean of antibody response per dose. Dotted lines: upper and lower specification limits calculated for GMU antibody response of aP Ctrl.

For testing of immunogenicity of the aP vaccine components, an IgG GMU assay was established according to European Pharmacopeia monograph^22^ to demonstrate consistency in guinea pig IgG responses against the different pertussis antigens contained in vaccine products from different manufacturers. A pediatric vaccine batch (aP Ctrl) was used as internal control. The aP Ctrl was first applied in three independent multi-dose assays (diluted 1/3 [D1], 1/9 [D2] and 1/27 [D3]) to prove dose-response (Supplementary Fig. 4). In the following experiments only the highest dose (1/3) was applied to guinea pigs (Fig. 5) to allow for setting up specifications at GMU mean ± 3 SD (log-transferred data; Fig. 5). These specification limits were subsequently applied to standardize and validate animal experiments with different DTaP and dtap vaccine batches containing different combinations and amounts of aP antigens (Fig. 5).

**Fig. 6:**
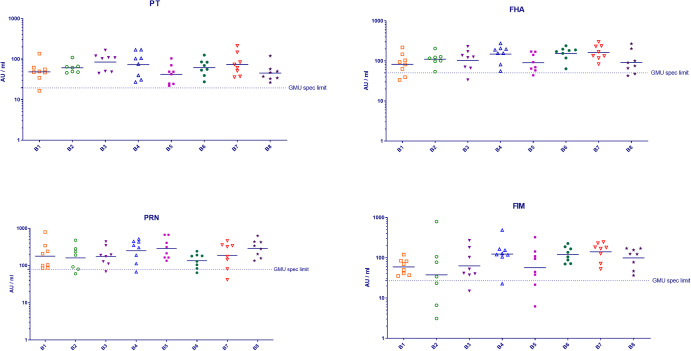
Immunogenicity of acellular pertussis components of various batches (B1 to B8) of dtap booster vaccine applied in separate animal studies at single-dose (1/1). Each symbol depicts the antibody response of one individual guinea pig against the respective aP antigen; bars: geomean of antibody response per batch (8 guinea pig sera/batch). Dotted line: specification limit calculated for GMU antibody response of dtap vaccine. Note that for batch B2 only 7 animals were available.

Similarly, aP GMU antibody specifications (GMU mean –3 SD of log-transferred data) were set up for every vaccine product (highest dose only) once data from sufficient batches were available. An example of aP antibody response against a dtap booster vaccine (vaccine dose D1 = undiluted) is provided in Fig. 6.

In case the GMU specification is not met for one or more of the aP IgG antibodies, the assay is to be repeated. Antibody responses of guinea pigs to aP vaccines varied to the same extent as those to diphtheria and tetanus toxoid components. However, by using the GMU assay this variability was leveled out and GMU IgG results for dtap vaccine batches were consistent for each of the four aP antigens.

### Immunization in the absence of vaccine components confirms method accuracy

The test results on potency of DTaP and dtap vaccine batches indicated that detection of the IgG response to the diphtheria component is critical because for some of the commercial vaccine batches diphtheria potency was calculated near the minimum amount required according to the European Pharmacopeia specification.

**Fig. 7:**
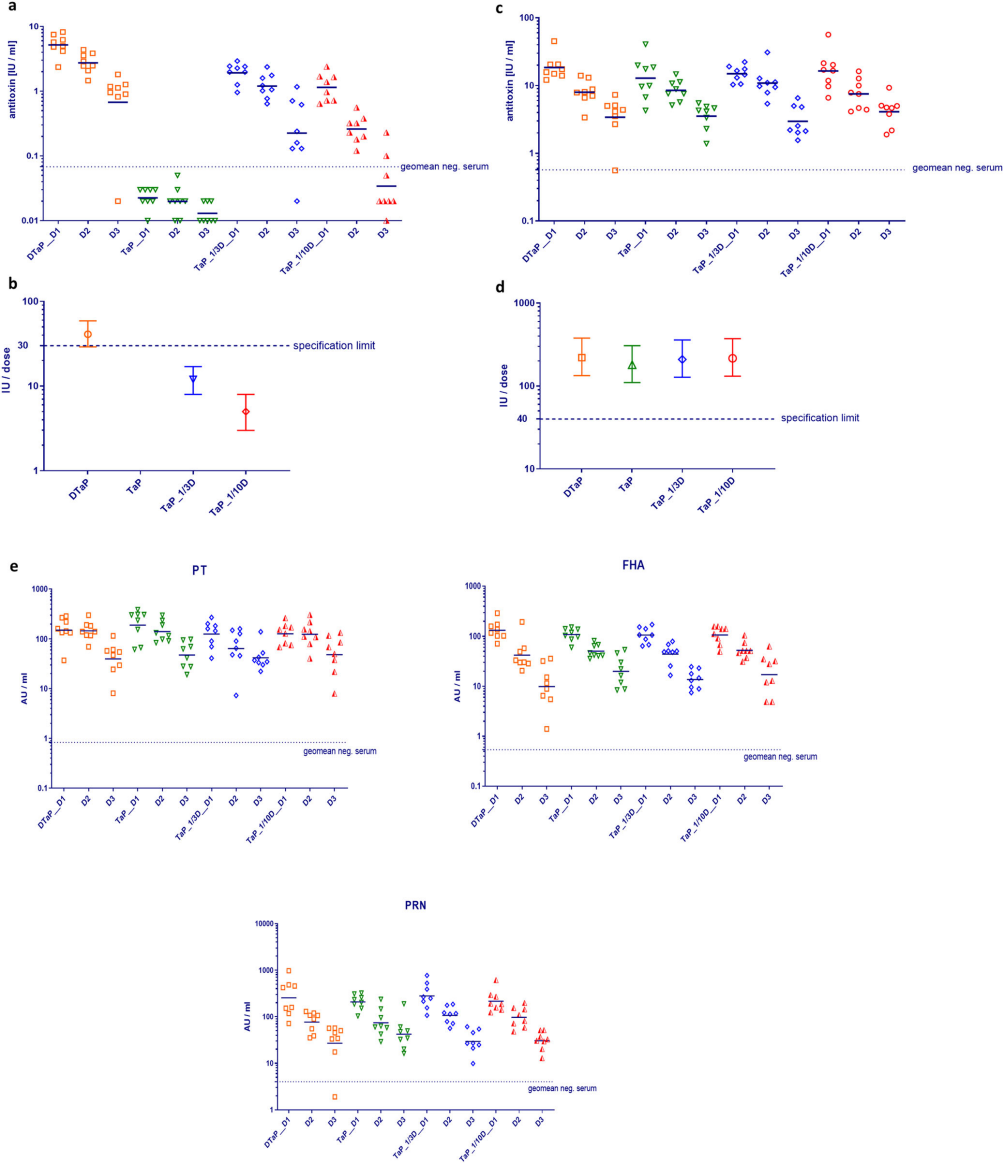
Immunogenicity of experimental pediatric vaccine batches containing complete (DTaP), 1/3 (TaP_1/3D) or 1/10 (TaP_1/10D) diphtheria dose or missing diphtheria component (TaP). All vaccines contained complete amounts of tetanus toxoid, pertussis antigens PT, FHA and PRN and adjuvant and were applied at 3 dose concentrations within the same animal study: D1 = 1/3, D2 = 1/9; D3 = 1/27. Immunogenicity of experimental vaccine batches against diphtheria (**a**), tetanus (**c**), and acellular pertussis (**e**). Each symbol depicts the IgG response of one individual guinea pig; bars: geomean of antibody response per dose. Dotted line: geomean of naïve guinea pigs (negative control serum). Diphtheria (**b**) and tetanus (**d**) potency (estimate ± 95% CI) obtained for experimental vaccines by calibration against 4th WHO IS Diphtheria Toxoid Adsorbed (07/216) and 4th WHO IS Tetanus Toxoid Adsorbed (08/218), respectively.

To evaluate method accuracy and precision of diphtheria potency determination, an experimental vaccine batch lacking the diphtheria toxoid component (TaP) was used with and without addition of indicated amounts of a regular DTaP vaccine batch. As a result, we obtained vaccine samples with 1/10 (TaP_1/10D) and 1/3 (TaP_1/3D) amount of diphtheria toxoid, while all experimental vaccine samples contained the regular amounts of tetanus toxoid (≥40 IU/dose) and pertussis antigens (25 µg each of PT and FHA, 8 µg of PRN) with all vaccine antigens adsorbed to an equal total amount of aluminum hydroxide (0.5 mg Al^3+^/dose). Groups of eight guinea pigs were immunized with equal doses of these experimental vaccine samples, TaP or the complete formulated DTaP vaccine, each diluted 1/3, 1/9 and 1/27 (Fig. 7).

The results showed that diphtheria antitoxin (IgG) levels in guinea pigs immunized with the DTaP complete vaccine (Fig. 7a) were in the range of those obtained with another pediatric vaccine with similar formulation (Fig. 4). By contrast, diphtheria IgG levels in guinea pigs immunized with TaP were in the range or below the geometric mean of the negative control serum, independent of the vaccine dilution/dose used for immunization (Fig. 7a). IgG responses of guinea pigs immunized with experimental vaccines containing diphtheria toxoid at 1/3 and 1/10 dose lay between those obtained with the regular DTaP and the TaP vaccine batches. Again, a clear dose-response relationship was observed. Moreover, calibration of diphtheria potency of experimental vaccine samples against the reference vaccine 4th WHO IS resulted in a potency estimate of 41 IU/dose (95% CI: 29–59 IU/dose) for DTaP, 12 IU/dose (95% CI: 8–17 IU/dose) for TaP_1/3D and 5 IU/dose (95% CI: 3–8 IU/dose) for TaP_1/10D, respectively (Fig. 7b), which is close to the potency that would have been calculated based on the dilution factors for D component, i.e., 14 IU/dose for TaP_1/3D and 4 IU/dose for TaP_1/10D compared to the potency of the complete DTaP vaccine. Of note, in this experiment and without repetition, the DTaP experimental vaccine batch failed the diphtheria potency test with a LL of 95% CI of 29 IU/dose (specification: ≥30 IU/dose).

The results obtained further showed that induction of antibody responses against tetanus and aP vaccine components were unaffected by the absence or reduction of the diphtheria content (Fig. 7c, e). Furthermore, tetanus potency estimates lay within a range of 181 – 220 IU/dose for each of the four experimental vaccines (Fig. 7d). The LL of 95% CI of tetanus potency of experimental vaccine batches lay between 110 and 134 IU/dose, which is far above the minimum amount requested for pediatric vaccines, i.e., 40 IU/dose. The data further showed that a dose-response relationship was obtained for each of the three aP antigens (PT, FHA, PRN) formulated in the experimental vaccine samples (Fig. 7e).

## Discussion

The guinea pig serology method for testing of DTaP combination vaccines was recently implemented in our batch release laboratory. Here we provide the protocol and the data that encouraged us to switch from challenge test to immunogenicity testing.

Although complete switching to in vitro release testing of vaccines would have been preferable, in vitro testing is difficult to realize for a variety of legacy vaccines produced by different manufacturers and tested worldwide^1,25,26^. Reduction and/or refinement of animal use in human vaccine potency testing can, however, reduce animal use and improve animal welfare worldwide while ensuring the continued safety and efficacy of human vaccines^27^. Notably, a 3 R International Workshop on Alternative Methods to Reduce, Refine, and Replace the Use of Animals in Vaccine Potency and Safety Testing held 2010 in Bethesda, Maryland, USA, identified complex vaccines containing diphtheria, tetanus and pertussis (acellular or whole cell) components as one of the highest priority human vaccines for development of reduction and/or refinement methods^3,27^.

While widespread efforts to establish product specific in vitro antigenicity assays for various DTaP/ dtap vaccine components have meanwhile been initiated^28–32^, serology techniques for concomitant quantification of serum antibodies against all DTaP vaccine antigens following immunization of animals have not adequately been driven forward^33^. The number of animals needed for potency testing could be reduced significantly by analysis of various antibodies in sera obtained from the same animals following immunization with DTaP or dtap combination vaccines^4,5,22^. Furthermore, animal distress is strongly reduced by applying serology tests instead of challenge tests^2^. The protocol described in this study bears the additional advantage that it can be used for testing of different antigens for multiple different products containing the diphtheria, tetanus and pertussis antigens. This reduces animal numbers and is, thus, in line with the 3 R roadmap. However, compared to challenge tests, use of serology-based methods prolongs testing periods because of additional in vitro testing after termination of the in vivo part.

Nevertheless, this does not exclude that continued efforts might abolish in vivo testing in accordance with the consistency approach that indirectly proves potency and safety through demonstration of preserved critical attributes of the vaccine formulation^34^. This will foster development and acceptance of cell-based assays for testing of functional integrity of vaccine antigens via induction of vaccine antigen-specific immune response^35,36^, ELISA-based methods detecting and quantifying immunogenic epitopes of antigens in vaccine formulations with well-characterized specific monoclonal antibodies^28–32,37,38^, or even non-invasive physical technologies such as Raman microspectroscopy^39^.

In this study multiplex ECLIA was used to analyze serum antibodies against diphtheria, tetanus and acellular pertussis antigens following inoculation of various doses of vaccines to guinea pigs, differing by a dilution factor of three. To quantify diphtheria and tetanus potency, additional groups of animals were immunized with 4^th^ WHO IS Diphtheria Toxoid and 4^th^ WHO IS Tetanus Toxoid at three different doses. As discussed previously, individual responses of animals were highly variable^7,8^. Nevertheless, a clear dose-response relationship between geometric mean antibody responses and vaccine dose was detected for every vaccine and reference preparation used. Furthermore, dose groups of 8 animals/dose for both test and reference vaccines were shown to be sufficient for a ‘pass’ result for diphtheria and tetanus potency of dtap booster vaccine batches and for tetanus potency of DTaP pediatric vaccine batches. However, DTaP vaccine batches occasionally did not pass the (first) diphtheria potency test. Repeat testing clearly showed that this ‘fail’ result followed from the variability of animal responses and did not indicate a diphtheria OOS result for this vaccine batch. Indeed, not any dtap or DTaP vaccine batch tested so far using the current serology assay finally failed the tetanus or diphtheria potency test in the combined assay following repeat testing. However, repeat testing generally results in prolonged testing periods, which can delay the release of vaccine batches for marketing purposes.

Total measurement uncertainty of ECLIA-based antibody measurement in guinea pig sera was estimated at or below 10%, which is deemed good when considering the complexity of the setup. Precision of results was in the range or better than those published for singleplex ELISAs^40–42^ and bead-based multiplex assays^9,12–15,20^. Furthermore, the enormous dynamic range of antibody quantitation over up to 4 logs scale using ECL detection represented a great advantage, because every single serum could be tested at the same starting dilution (1:400), thus avoiding repeat tests of sera with very low or very high IgG concentrations.

To date, immunogenicity determination is already the method of choice to determine potency of acellular pertussis vaccines. However, testing is performed in mice, whereas, in the present setup, antibodies against aP vaccine components were quantified in sera obtained from guinea pigs. Unfortunately, there is no reference preparation available for analysis of pertussis antibodies in guinea pig sera. Thus, in-house standard and positive control serum were generated using pools of guinea pig sera following immunization with dtap vaccines containing the aP antigens PT, FHA, PRN and FIM. The positive control serum was assigned 100 AU/ml for each anti-pertussis antigen IgG and used to calibrate the pertussis antibodies in standard serum in a series of 72 tests.

To demonstrate that OOS vaccine batches are detected using guinea pig serology combined with ECLIA serum analysis, an experimental batch of a pediatric diphtheria drop-out vaccine formulated without any diphtheria toxoid (TaP) but with full amounts of tetanus and acellular pertussis antigens plus aluminum hydroxide adjuvant (0.5 mg Al^3+^/dose) was analyzed in a multi-dose assay in guinea pigs. The TaP batch was compared to a vaccine batch formulated with full diphtheria toxoid amount (DTaP) and mixtures of both resulting in batches with reduced diphtheria toxoid doses that clearly failed the diphtheria potency test, whereas potency of the DTaP batch was estimated at 41 IU/dose which is in the range of other pediatric vaccine batches (Fig. 4b). The LL of 95% CI was calculated at 29 IU/dose, i.e., formally the European Pharmacopeia specification of ≥30 IU/dose was not met using the complete formulated DTaP vaccine^24^, indicating an inherent weakness of the test. However, as shown before, in the background of variability in animal antibody responses this is not unusual.

It should be mentioned that tetanus potency and aP immunogenicity results of all experimental vaccines were in the same range showing that formulation of vaccine batches with differing amounts of diphtheria toxoid but same composition of other vaccine antigens and adjuvant does not affect the immunogenicity of the other vaccine components. It should further be noted that all experimental vaccines contained same amount of aluminum adjuvant (0.5 mg Al^3+^) which is known to potentiate immunological responses^43–45^. Thus, the reduction in diphtheria potency of TaP_1/3D and TaP_1/10D experimental vaccines was clearly attributable to the reduced diphtheria toxoid content and not caused by changes in adjuvant content as occurs by vaccine dilution.

In summary, we developed a high-throughput ECLIA for specific IgG quantitation in sera from immunized guinea pigs for simultaneous potency determination of diphtheria, tetanus and acellular pertussis components in combination vaccine batches. The assay discriminates between diphtheria in-specification and diphtheria OOS batches, and, has the potential to be routinely applied for dtap and DTaP vaccine potency testing in governmental batch release testing. In the framework of 3 R animal welfare, serology-based assays provide significant potential of refinement. Our data suggest that serological potency determination can be reasonably applied until validated in vitro tests systems are available for vaccine products and product components used worldwide for prevention of disease caused by *Corynebacterium diphtheriae*, *Clostridium tetani*, and *Bordetella pertussis*.

## Methods

### In vivo immunization experiments

In vivo immunization studies were performed in guinea pigs in accordance with the respective European Pharmacopeia assay monographs for diphtheria vaccine (method C), tetanus vaccine (method C), and pertussis vaccine (acellular, method B)^4,5,22^. The use of laboratory animals for these studies was approved by the Regierungspraesidium Darmstadt (approval F107/1014 and F107/1023).

Female Dunkin Hartley guinea pigs were obtained from Charles River Sulzfeld, Germany with a body weight of 250–300 g. Animals were randomly allocated to groups of 8 guinea pigs/vaccine dose. Infrequently, single guinea pigs provided by the supplier could not be included in the animal experiment because of disease or fractured or misaligned incisors. In this case, all residual healthy animals were evenly allocated to dose groups (at least 7 guinea pigs/dose). Following an acclimatization period of 6 to 8 days, guinea pigs were inoculated subcutaneously with 1 ml of vaccine or reference dilution. Up to 8 batches of 2 representative vaccines from different manufacturers were applied: (1) a booster dtap vaccine from manufacturer A formulated using 2 IU diphtheria toxoid, 20 IU tetanus toxoid, 2.5 µg pertussis toxoid (PT), 5 µg each of filamentous hemagglutinin (FHA) and fimbriae proteins type 2/3 (FIM), and 3 µg pertactin (PRN) per single human dose (SHD), and (2) a pediatric DTaP-HB-IPV vaccine from manufacturer B formulated using 30 IU diphtheria toxoid, 40 IU tetanus toxoid, 25 µg each of PT and FHA and 8 µg PRN per SHD. In addition, the latter contains 10 µg hepatitis B (HB) antigen and 40, 8, and 32 D-antigen units of inactivated polio virus (IPV) types 1, 2, and 3, respectively, per SHD. It should be noted, however, that immune responses against HB and polio virus antigens were not analyzed in the present study. For each vaccine batch, three doses diluted using sterile 0.9% sodium chloride solution were applied^4,5,22^. Pediatric vaccine batches were applied at dilutions 1/3, 1/9, 1/27, booster vaccine batches were used at dilutions of 1/1 (undiluted), 1/3, 1/9.

For calibration of D and T potency of vaccines, additional groups of animals (8/dose) received 1 ml/dose reference vaccine preparation. 4^th^ World Health Organization (WHO) International Standard (IS) for Diphtheria Toxoid Adsorbed (No. 07/216, NIBSC, UK) was applied at doses 28.5, 9.5 and 3.17 IU/ml and 4^th^ WHO IS for Tetanus Toxoid Adsorbed (No. 08/218, NIBSC, UK) applied at doses of 66, 22, and 7.33 IU/ml. Both reference vaccines, at corresponding doses (high, medium or low), were applied to the same animals at different body sides to reduce the number of animals used per experiment.

As internal control for acellular pertussis immunogenicity, a pediatric vaccine batch (aP Ctrl) containing the pertussis antigens PT (10 µg/SHD), FHA (5 µg/SHD), PRN (3 µg/SHD) and FIM (5 µg/SHD) was qualified in-house as described in Methods section ‘determination of acellular pertussis immunogenicity’. For routine testing, aP Ctrl was applied to another group of 8 animals at a single dilution of 1/3. Initially, however, 3 dilutions of aP Ctrl were applied to check dose-response in 3 animal studies and thereby confirm suitability of aP Ctrl as internal control. Blood collection for the detection of serum antibodies (endpoint) was performed 35 days after immunization. To this end, guinea pigs were narcotized using 45 mg ketamine/ kg body weight plus 2.5 mg xylazine/ kg body weight (i.m.). When animals have attained deep anesthesia status, blood was drawn via heart puncture and collected in serum tubes with clot activator. Animals were euthanized via cervical dislocation while still in deep anesthesia. After 1 h at room temperature (RT), blood tubes were centrifuged for 10 min at 2500 × *g*, serum was harvested and stored at -20 °C until analysis.

### Immunization with subpotent doses of vaccine antigen

To prove the ability of the guinea pig-based serology assay to detect subpotent batches of diphtheria vaccines, a diphtheria dropout experiment was carried out using experimental pediatric vaccine batches formulated with (DTaP) or without (TaP) diphtheria component (kindly provided by GSK, Rixensart, Belgium). Both experimental vaccines contained full amounts of tetanus toxoid (at least 40 IU/SHD) as well as three acellular pertussis antigens PT (25 µg/SHD), FHA (25 µg/SHD), and PRN (8 µg/SHD), but no FIM antigens. Furthermore, all experimental batches were formulated using the same amount of aluminum hydroxide, i.e., 0.5 mg Al^3+^/SHD. These vaccines were mixed to obtain experimental vaccines containing diphtheria toxoid at a dose of 1/3 or 1/10 (TaP_1/3D, TaP_1/10D), together with full amounts of T and aP components. All 4 experimental vaccines were then used for immunization of guinea pigs at doses of 1/3, 1/9 and 1/27. Seraobtained 35 days after immunization were analyzed in the multiplex assay as follows.

### Multiplex assay for antibody quantification in guinea pig sera

To quantify the vaccine antigen-specific serum antibodies, we established a hexaplex assay based on U-plex development pack (6-assay) from Mesoscale Discovery (MSD, Rockville, USA). The U-plex 96-well plates are provided with six different linkers that allow binding of six different biotinylated proteins to linker-specific, precoated sites (spots) on the bottom of each well (Supplementary Fig. 1a). Antigens were biotinylated and purified using the EZ-Link^TM^ Sulfo-NHS-LC-Biotinylation kit (Thermo Fisher Scientific, Dreieich, Germany) according to the manufacturer´s instructions. The following antigens were used: diphtheria toxin (unnicked, DTx) (Merck-Aldrich, Darmstadt, Germany), tetanus toxin (TTx) (List Biological Laboratories, Campbell, USA), pertussis toxin (PTx), FHA, FIM (Sanofi Pasteur, Toronto, Canada), and PRN (GSK, Rixensart, Belgium). In accordance with the instructions provided, biotinylated proteins were incubated separately with one of the six linkers (Supplementary Fig. 1a), followed by addition and incubation with stop solution and mixture of all antigen-linker solutions at equal volumes to a master mix, which was subsequently applied into the assay plate (50 µl/well). The optimum amount of each of the biotinylated antigens used for linker coupling was established in pilot experiments to maximize the difference of ECL values between positive (i.e., vaccinated) and negative (i.e., naïve) guinea pig sera. To exclude potential impact of cross-reaction of serum antibodies with unrelated antigens, a series of singleplex assays were carried out using individual antigens bound to their respective linker, but in the presence of five residual uncoupled linkers treated and ‘inactivated’ in the same way as the linker coupled to the respective antigen. Singleplex assay dilution curves obtained with standard, positive and negative control sera were compared to those of multiplex assay dilution curves.

Following binding of master mix antigens (1 h at RT on a plate shaker at 700 rpm), assay plates were washed three times with wash buffer (0.5% Tween 20 in phosphate-buffered saline (PBS, Ca^2+^ and Mg^2+^ free)), followed by incubation with serially diluted guinea pig sera for 1 h at RT on a plate shaker at 700 rpm. During the incubation period specific antibodies bound to the respective biotinylated protein (vaccine antigen) and captured immunoglobulins (IgG) are subsequently detected with Sulfo-TAG-labeled donkey-anti-guinea pig IgG F(ab)_2_ fragments (Supplementary Fig. 1b). Notably, sera were diluted 1:400, then sequentially diluted fourfold in 6 steps (total of 7 dilutions) with SuperBlock^TM^ (PBS) Blocking Buffer (Thermo Fisher Scientific, Dreieich, Germany) in a non-binding microtiter plate (Greiner Bio-One, Frickenhausen, Germany) and finally transferred into the assay plate (50 µl/well). Each plate was incubated with a standard serum (applied in duplicate dilution lines) as well as a positive and a negative control - plus up to eight individual test sera (all in single dilution line) at the same serial dilution.

Following three times washing with wash buffer, U-plex plates were incubated with 50 µl/well of Sulfo-TAG-labeled donkey-anti guinea pig IgG F(ab)_2_ fragment (Jackson ImmunoResearch, West Grove, USA; #706-006-148) as secondary antibody diluted 1:1400 with SuperBlock^TM^ (PBS) Blocking Buffer for 1 h at RT on a plate shaker (700 rpm). Labeling and purification of secondary antibody was performed using MSD Gold Sulfo-TAG NHS-Ester according to instructions of the manufacturer (MSD). Subsequently, plates were washed three times with wash buffer and filled with 150 µl/well of MSD Gold Read Buffer B (MSD, provided with U-plex kit). Detection of IgG concentrations was based on ECL measurement using the MESO Quickplex SQ 120 instrument equipped with Methodical Mind Plate Reader software and a cooled CCD camera (all from MSD).

Plates were measured under voltage connection and ECL signals at each spot were detected by means of a high-resolution camera. The technique decouples the stimulation (electricity) from the signal (light emission) so that only labels near the electrode surface are detected, which results in a very low background signal but maximum signal-to-noise ratio. Furthermore, multiple excitation cycles are used at each spot to amplify the signal, allowing for ultra-sensitive detection and a broad dynamic range, i.e., low to high IgG levels can be quantified within the same plate, covering an ECL scale of up to 4 logs.

### Generation of control sera for standardization

Standard and positive control sera were qualified internally using pools of sera obtained from 72 and 17, respectively, guinea pigs immunized with dtap (small letters indicate low antigen) vaccine batches. Accordingly, negative serum was generated by pooling serum from 14 non-immunized (naïve) guinea pigs. Quantitation of anti-diphtheria and anti-tetanus antibodies in guinea pig standard and positive control sera was performed by calibration in a total of 72 tests against Reference Material Diphtheria and Tetanus Antitoxin (No. 98/572, NIBSC, UK; containing 3 IU/ml diphtheria antitoxin and 3.5 IU/ml tetanus antitoxin). Since no guinea pig reference serum against pertussis antigens is available, pertussis antibodies are quantified in test sera using arbitrary units (AU) based on an assigned concentration of 100 AU/ml of the control serum for each pertussis antibody, i.e., IgG against antigens PTx, FHA, PRN and FIM. The anti-pertussis IgG concentration in standard serum was calibrated in a series of overall 72 tests against positive control serum.

Because of the huge signal amplification in the ECLIA at lower dilutions, negative control serum also displayed a concentration-dependent background response, which allowed calculation of ‘virtual’ antibody concentrations in negative serum. Thus, virtual antibody concentrations were calculated by calibration against the standard in a series of 72 assays. For this purpose, ECL results obtained for the three highest negative serum concentrations were calibrated against those of the standard accepting deviations from linearity and/or parallelism.

Using the results obtained for positive and negative serum in 72 tests, specification limits were calculated for positive and negative controls according to the following formulae using log-transformed data:

$${\rm{Upper\; specification\; limit}}:\exp \left({mean}+3* {SD}\right)$$

$${\rm{Lower}}\; {\rm{specification}}\; {\rm{limit}}\,({\rm{positive}}\; {\rm{control}}\; {\rm{only}}):\exp \left({mean}-3* {SD}\right)$$

where mean is the arithmetric mean and SD is standard deviation of log-scaled antibody concentrations.

### Quantification of IgG concentrations

Quantitation of IgG concentrations in test and control sera was performed by calibration of ECL values against those of standard serum on the same plate using CombiStats™ software (European Directorate for the Quality of Medicines and HealthCare, Strasbourg, France) and parallel line model (European Pharmacopeia 2023 g). Validity criteria for each plate and each antigen are (1) positive control serum within its lower and upper specification limit and (2) negative control serum below its (upper) specification limit.

### Assay characterization and specifications

The precision of the multiplex assay was assessed via determination of intra-assay and inter-assay variation. To calculate the total as well as intra-assay and inter-assay coefficient of variation, we used antibody values obtained from positive control serum (against 6 antigens) from 20 assays (17 assays with four plates in parallel and 4 assays with one plate) all performed using standard conditions by two technicians on different days. An Analysis of Variance model (ANOVA) was applied (separately for each IgG antibody) to the logarithmized data with ‘day’ and ‘operator’ as random effects. The total variance estimated form the ANOVA model was used for the measurement uncertainty, the sum of ‘day’ and ‘operator’ variance as estimate for inter-assay variability, and the residual variance as estimate for intra-assay variability. Evaluation was performed using SAS®/STAT Software, Version 9.4 (procedure PROC MIXED). Assay specificity was determined indirectly by accordance of ECL values with expected values in vaccine groups missing one or more pertussis antigens. I.e., sera from dtap vaccine groups (S1) are compared to sera from the diphtheria and tetanus reference vaccine groups (S2) and sera obtained following immunization with DTaP-HB.IPV vaccines comprising PT, FHA, and PRN but no FIM (S3).

### Diphtheria and tetanus potency determination

Diphtheria and tetanus potency (estimate and 95% confidence interval [CI]) of test vaccine batches was calculated by means of CombiStats in IU/0.5 ml ( = 1 SHD) relative to 4^th^ WHO IS Diphtheria Toxoid Adsorbed or 4th WHO IS Tetanus Toxoid Adsorbed, respectively, using the IgG concentration of each of the eight guinea pig sera per test vaccine or reference vaccine dose. The potency is calculated based on individual antibody titers in each dose group. Generally, log- or square root-transformed IgG concentrations were used for potency calculation to fulfill conditions of linearity and parallelism^46^.

Potency values were rounded to whole numbers of IU/dose^47^. Note that according to the European Pharmacopeia, a vaccine batch meets the requirement for diphtheria potency if it contains at least 2 IU/dose diphtheria toxoid in case of booster vaccines or 30 IU/dose in case of pediatric vaccines at the lower limit [LL] of 95% CI. The vaccine batch meets the requirement for tetanus potency if at least 20 IU/dose tetanus toxoid was calculated in case of booster vaccines or 40 IU/dose in case of pediatric vaccines (LL of 95% CI). If a vaccine batch does not meet the respective requirements, the animal test has to be repeated once or twice (if necessary) and results of all tests are to be analyzed in a combined assay using CombiStats™, taking into account the homogeneity of the results (for details see results section).

### Determination of acellular pertussis immunogenicity

aP immunogenicity was determined with the highest vaccine dose group only (1/3 for pediatric vaccine, 1/1 for booster vaccine). However, in accordance to European Pharmacopeia recommendation^22^, both the in-house reference vaccine (aP Ctrl) and all vaccine products at first were analyzed in at least three multiple-dose assays to demonstrate dose-related antibody responses in guinea pigs. Following confirmation of dose-response relationship, aP immunogenicity of further batches was then analyzed in the highest dose group per vaccine product only. Logarithm results of geometric mean units (GMU)/ml serum obtained in at least 10 animal assays per vaccine (same batch used for aP Ctrl, different batches for vaccine products) were used to calculate in-house specification limits for each product according to following formulae:

$${\rm{Lower\; limit}}:{10}^{\left({mean}-3* {SD}\right)}$$

$${\rm{Upper}}\; {\rm{limit}}\,({aP\; Ctrl\; only}):{10}^{\left({mean}+3* {SD}\right)}$$

where mean is the arithmetric mean and SD is standard deviation of log-scaled GMU antibody concentrations.

### Supplementary information

Supplementary Information

## Data Availability

All data from this study are available from the corresponding author upon reasonable request.
