# Supplementary material for: Determination of DTaP vaccine potency by multiplex immunogenicity testing using electrochemiluminescence
Source: NPJ Vaccines. 2024 Aug 7;9:142. doi: 10.1038/s41541-024-00915-y (PMC11306252; doi:10.1038/s41541-024-00915-y)
Supplement: Supplementary file 1 — Supplementary Information [file 41541_2024_915_MOESM1_ESM.pdf]

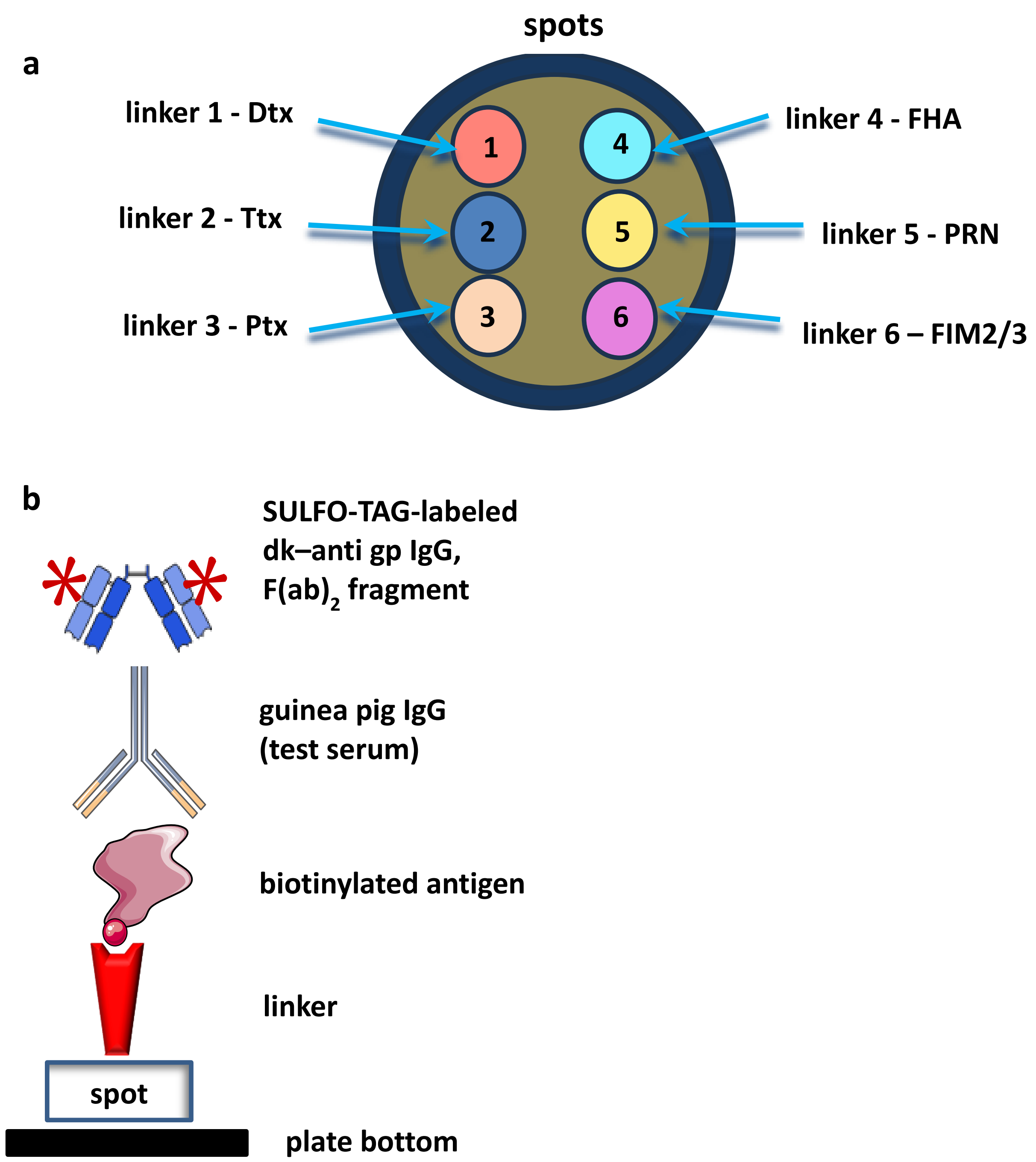

Supplementary Figure 1

a; schematic representation of hexaplex assay using MSD U-plex development pack with 6 spots activated (spots 1, 2, 3, 8, 9, 10). Respective linkers coupled to individual biotinylated antigens bind to corresponding spots, thereby fixing the antigens to the plate bottom.

b; schematic representation of ECLIA using one spot of U-plex plate. Biotinylated antigen is bound to the plate spot via respective linker. In a second step, guinea pig serum IgG bind to respective plate-bound antigen. Guinea pig antibodies are then detected by secondary antibodies (donkey-anti guinea pig IgG, $F(ab)_2$ fragment) labeled with Sulfo-TAG. The amount of Sulfo-TAG label is proportional to the electrochemiluminescence signal induced by electricity applied to plate electrodes.

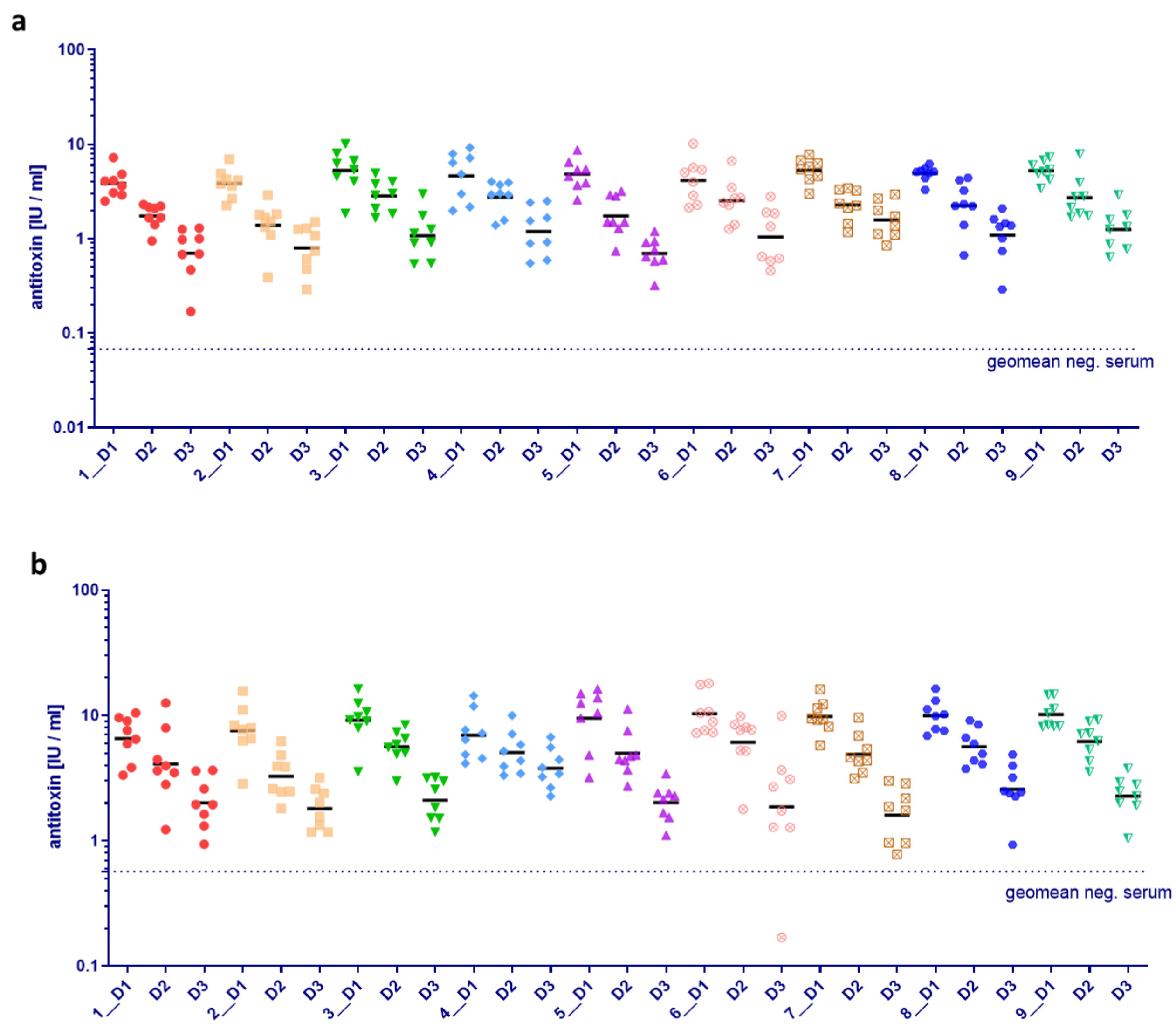

Supplementary Figure 2

Immunogenicity of 4th WHO IS Diphtheria Toxoid Adsorbed (07/216, **a**) and 4th WHO IS Tetanus Toxoid Adsorbed (08/218, **b**) in guinea pigs. Vaccine preparations were applied at 3 dose concentrations in nine separate animal studies (1-9): Diphtheria Toxoid Adsorbed: D1 = 28.5 IU/ml; D2 = 9.5 IU/ml; D3 = 3.17 IU/ml; Tetanus Toxoid Adsorbed: D1 = 66 IU/ml; D2 = 22 IU/ml; D3 = 7.33 IU/ml. Each symbol depicts the antibody/antitoxin response of one individual guinea pig; bars: geomean of antibody response per dose. Dotted line: geomean of naïve guinea pigs (negative control serum).

a

b

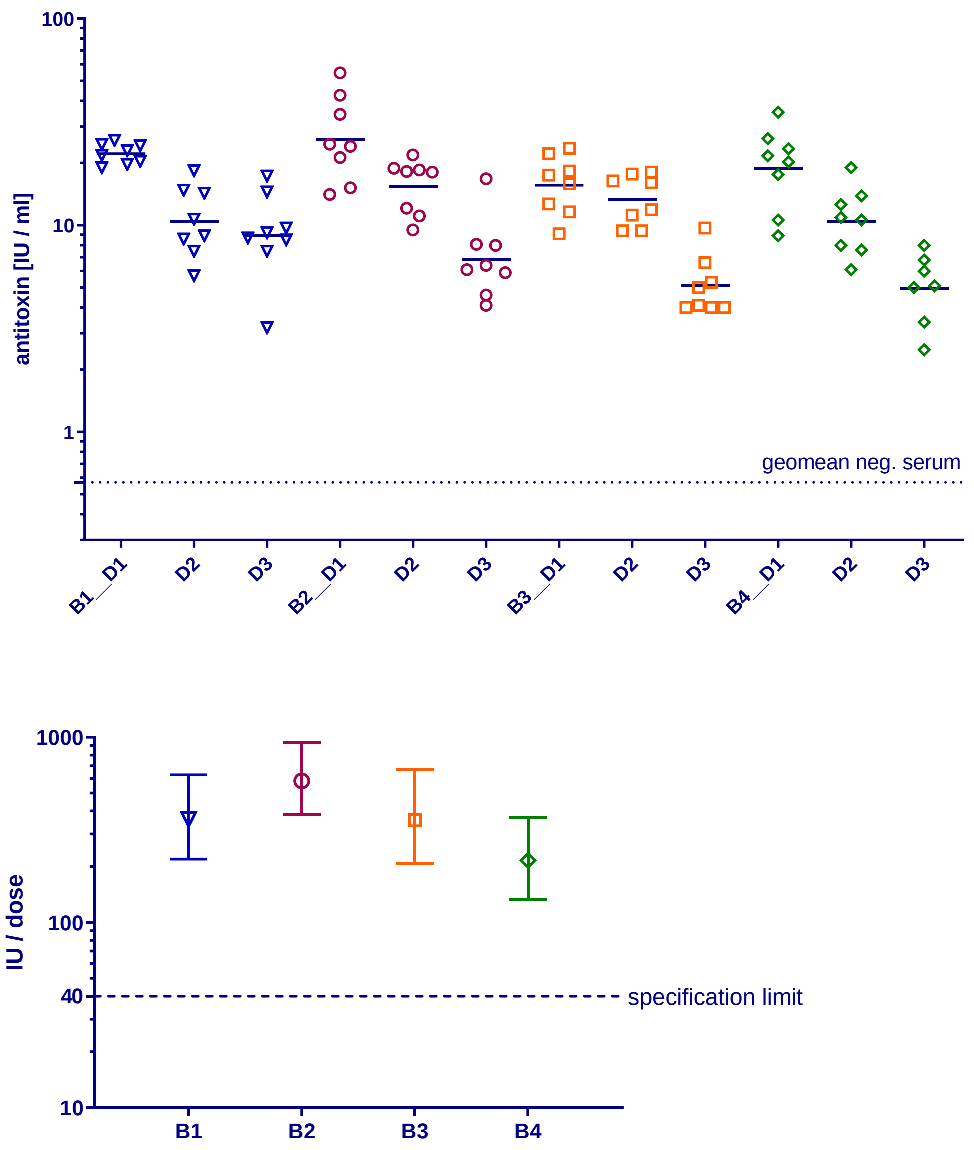

Supplementary Figure 3

a; Immunogenicity of tetanus component of four batches of a DTaP pediatric vaccine in guinea pigs. Vaccine batches (B1 to B4) were applied at 3 dose concentrations. D1 = 1/3, D2 =1/9, D3 = 1/27. Each symbol depicts the IgG/antitoxin response of one individual guinea pig; bars: geomean of antibody response per dose. Dotted line: geomean of naïve guinea pigs (negative control serum). Note that for dose group B4a_D3 only 7 animals were available.

b; Tetanus potency (estimate ± 95% CI) obtained for DTaP pediatric vaccine batches by calibration against 4th WHO IS Tetanus Toxoid Adsorbed (08/218). For all batches a tetanus toxoid content above the minimum amount of 40 IU/dose at lower limit of 95% CI was calculated, i.e., the batches ‘passed’ tetanus potency test.

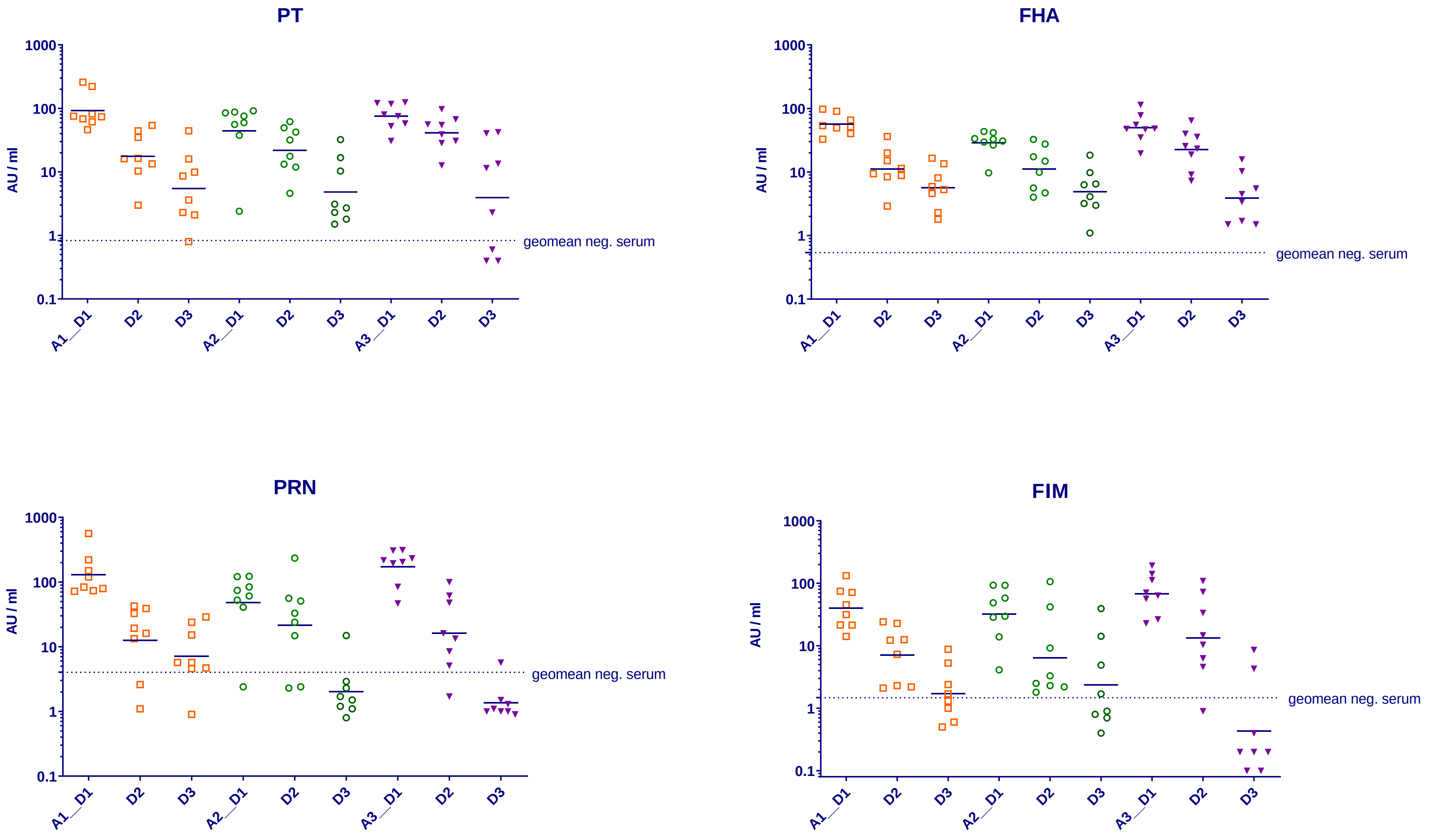

Supplementary Figure 4

Immunogenicity of acellular pertussis components of aP Ctrl vaccine batch applied in separate animal studies (A1 to A3) at multi doses. aP Ctrl applied at doses D1 = 1/3, D2 =1/9, D3 = 1/27. Each symbol depicts the antibody response of one individual guinea pig against the respective aP antigen; bars: geomean of antibody response per dose. Dotted line: geomean of naïve guinea pigs (negative control serum).
